# Intrusion detection in software defined network using deep learning approaches

**DOI:** 10.1038/s41598-024-79001-1

**Published:** 2024-11-25

**Authors:** M. Sami Ataa, Eman E. Sanad, Reda A. El-khoribi

**Affiliations:** Fuclty of Computers and Artificial Intelligence, Cairo University, Giza, Egypt

**Keywords:** Energy science and technology, Engineering, Mathematics and computing

## Abstract

Ensuring robust network security is crucial in the context of Software-Defined Networking(SDN). Which, becomes a multi-billion dollar industry, and it’s deployed in many data centers nowadays. The new technology provides network programmability, network centralized control, and a global view of the network. But, unfortunately, it comes with new vulnerabilities, and new attack vectors compared to the traditional network. SDN network cybersecurity became a trending research topic due to the hype of Machine Learning (ML) when a group of Machine Learning(ML) techniques called Deep Learning(DL) started to take shape in the setting of SDN networks. This paper focuses on developing advanced Deep Learning(DL) models to address the inherent new attack vectors. In this paper, we have built and compared two models that can be used for building a complete Intrusion Detection System(IDS) solution, one using a hybrid CNN-LSTM architecture and the other using Transformer encoder-only architecture. We specifically target the SDN controller where it represents a crucial point. We utilized the InSDN dataset for training and testing our models, this dataset captures real-world traffic within the SDN environment. For evaluation, we have used accuracy, precision, recall, and F1 Score. Our experiment results show that the Transformer model with 48 features achieves the highest accuracy at 99.02%, while the CNN-LSTM model achieves 99.01%. We have reduced the features to 6 and 4, which gave us varying impacts on the models’ performance. We have merged 4 poorly represented attacks in one class, which enhanced the accuracy by a significant score. Additionally, we investigate binary classification by merging all attack types into a single class, as a result, the accuracy increased for both models. The CNN-LSTM model achieves the best results with an accuracy of 99.19% for 6 feature sets, this enhances the state-of-the-art results.

## Introduction

Keeping track of network security is a major concern in modern communication networks. SDN provides a new architecture for the network, which separates the data forwarding functions from control logical functions. SDN enables dynamic changes to thread policies and facilitates network programmability^1^. Also, it manages its operators to develop different applications by making use of the new logically centralized control view of the network, and this have increased the flexibility and programmability of the network. Unfortunately, the new architecture brings new attack vectors^2^, notably in the SDN controller^3^. Securing such architecture requires addressing several aspects, one of these points is the SDN controller which serves as the brain of the SDN network^4^ and the primary concern of this paper.

To enhance overall SDN network security for future deployments, we must address all the security issues that came up with the new architecture^5^. One of the key strategies for preventing cyber-attacks is the prompt identification of the attack process using an intrusion detection system (IDS). A network intrusion detection system (IDS) is employed to identify unauthorized activities on a digital network^6^. Due to the programmability of the SDN, we can develop the IDS to work as an SDN application. There are primarily two different types of IDS: signature-based IDS, and anomaly-based IDS. The main difference between these systems is the ability of anomaly-based to detect unknown cyber-attacks, whereas signature-based can only identify well-known patterns existing in the system.

Machine Learning (ML) contains significant techniques that can be applied within SDN architecture to enhance both functional and non-functional aspects of the network, such as security, performance, and others^7^. Deep learning (a subset of Machine Learning) models have the ability to deal with large-scale data and it demonstrated success across various fields^8^. However, it is difficult to improve network IDS with ML/DL techniques, because it requires train and assessing the model with correctly labeled normal and malicious samples^9^. Researchers utilizing ML/DL-based IDS in SDN networks have found that the scalability of both SDN and ML/DL algorithms has significantly improved IDS accuracy^10^. Machine Learning (ML) and Deep Learning (DL) approaches show the power of modern algorithms in detecting patterns and anomalies that traditional methods might miss. A well-trained ML/DL model can distinguish between normal and malicious traffic, which provides a robust defense mechanism against cyber threats. Various ML/DL techniques such as Naïve Bayes, Support Vector Machine (SVM), Logistic Regression, Neural Networks (NN), Convolutional Neural Networks (CNN), Recurrence Neural Networks (RNN), Long-Short Term Memory (LSTM), and lately Transformers can be used to build effective models.

In this paper, we have created two DL models for constructing intrusion detection systems, utilizing state-of-the-art techniques to enhance detection accuracy and reduce false alarm rates. We evaluated our models’ performance using accuracy, precision, recall, and F1 score.

To the best of our knowledge, we are the pioneers in utilizing the Transformer model along with the InSDN dataset for SDN security. The rest of this paper is as follows. Section 2 will cover related works. Section 3 provides a brief background on LSTM and Transformers. Section 4 introduces our models’ architectures. Section 5 presents and compares the results obtained. Section 6 contains the conclusion of the paper. Finally, Sect. 7 outlines potential future work.

## Related works

This study focuses on deep learning (DL) approaches, abandoning algorithms such as K-Nearest Neighbors (KNN), Support Vector Machine (SVM), Logistic Regression (LR), Decision Trees (DTs), and Naive Bayes (NB) classifiers. Because all these algorithms rely on pre-defined features can limit the models’ capabilities and are known for their high false alarm rates^11^. In contrast, DL does not require any feature extraction. It is capable of investigating raw data deep structures and automatically extracting relationships between different data records. Over the past years, DL techniques have been widely used for building Intrusion Detection System (IDS) models. This section introduces the most recent DL approaches for that purpose.

Tuan Anh Tang et al.^12^ employed a Gated Recurrent Unit Recurrent Neural Network (GRU-RNN) they tested their model using NSL-KDD and CICIDS2017 datasets with a classification accuracy of 89% and 99% respectively. Both datasets are considered old and based on traditional networks, which don’t represent SDN modern networks fairly.

S. S. Volkov et al.^13^ used Long Short-Term Memory (LSTM) based neural networks to build their model. They used the CSE-CICIDS2018 dataset for training and testing. However, CSE-CICIDS2018 contains several different attack scenarios such as Brute force, Botnet, web-attack, Dos, and DDos. It was also generated from traditional network flows.

A. Soliman et al.^14^ introduced models relays on Recurrent Neural Networks (RNN), Long Short-Term Memory (LSTM), and Gated Recurrent Unit (GRU). They used the InSDN dataset for training and testing, they tested their models using two different feature sets one including 48 features which achieved accuracies between 98.0% and 98.85 and another including 6 features that achieved accuracies range between 91.1 and 92.5%.

Wang et al.^15^ designed a hybrid structure called DDosTC, that combines the Transformers attention technique and Convolution Neural Network (CNN) to detect distributed denial-of-service (DDoS) attack on SDN, the model was tested and evaluated using the most recent CICDoS2019 dataset.

M. S. Elsayed et al.^11^ developed another hybrid model that combines Convolutional Neural Network (CNN) and Long Short-Term Memory (LSTM) this time. They achieved an accuracy of 96.32% on the InSDN dataset.

Zihan Wu et al.^16^ formulated a robust Transformer-based Intrusion Detection System (RTIDS). The model utilizes positional embedding to capture sequential information between features, followed by a stacked encoder-decoder neural network to learn low-dimensional features. In addition to a self-attention mechanism to improve classification. They demonstrate the effectiveness of RTIDS on the CICID2017 and CICDDoS2019 datasets, achieving F1-Scores of 99.17% and 98.48% respectively.

In Z Long et al.^17^ a simple encoder-decoder Transformer was used for classifying CICIDS2018 attacks, the model achieved an F1-score of 93.9% and 48.9% for DoS AND DDoS respectively.

V. Hnamte et al.^18^ used Convolution Neural Networks (CNN) along with Bidirectional Long-short Memory (BiLSTM), to create a model which achieves 100% and 99.64% accuracy rates on CICIDS2018 and Edge_IIoT datasets, respectively, according to their experiments.

Ivandro O. Lopes et al.^19^ designed and implemented a set of temporal-based convolutional models. They evaluated their models against CICDDoS2019 and CSE-CIC-IDS2018 datasets. Their models achieved performance in the range between 98.07% and 99.95% for the majority of considered metrics, according to their experiments.

Ganesh Khekare et al.^20^ propose a hybrid Generative Adversarial Network-Recurrent Neural Network (GAN-RNN) model. The model consists of two parts, a GAN architecture that creates custom synthetic traffic patterns that are used for enhancing security analysis, and an RNN for predicting the upcoming network attacks. The CICIDS2017 dataset achieved 99.4% for both accuracy and F1-score metrics.

A. Meliboev et al.^21^ tried several models, including Convolutional Neural Network (CNN), Long-Short Term Memory (LSTM), Gated Recurrent Unit (GRU), Recurrent Neural Network, and CNN-LSTM model. They used The KDD_cup99, NSL-KDD, and UNSW_NB15 datasets were used to evaluate the proposed models. The lowest accuracy was observed in RNN with UNSW_NB15 (71.9%), and the highest was for CNN with KDD_cup99 (95.2%).

V. Hnamte et al.^22^ proposed a deep learning hybridized technique that consists of two stages, Long-Short Term Memory (LSTM) and Auto-Encoder (AE). Performance was evaluated using CICIDS2017 and CSE-CICIS2018 datasets which achieved 99.99% and 99.1% accuracy, respectively.

M. Mahmoud et al.^23^ employed an Autoencoder combined with LSTM to perform intrusion detection in an IoT environment. They used the NSL-KDD dataset and achieved 98.88% accuracy. The authors utilized only Standard Scaling and not any other preprocessing steps.

A. S. Issa et al.^24^ proposed a distributed denial-of-service (DDoS) attack mitigator based on the CNN-LSTM model. The NSL-KDD dataset was also used here for training and testing the model, they achieved 99.02% accuracy. However, due to the dataset’s age, we can’t rely on it for detecting modern network attacks.

James Dzisi et al.^25^ proposed a hybrid model that consists of RNN and LSTM for mitigating DDoS attacks on SDN controllers. The authors built their dataset using a virtual environment. They investigate various split ratios 82/20, 70/30, and 60/40. Their best accuracy was 89.63% achieved using a 70/30 split ratio.

T Zhang et al.^26^ introduce a Transformer-based model that mitigates the relay link forgery attack. The authors built their dataset using a simulated environment, the environment was built using Mininet and the Ryu controller. After a month of collecting data, they end up with 334,000 records. Their model achieves around 96.5% accuracy and 96.2% F1 score.

R. A. Elsayed et al.^27^ built a two-level intrusion detection system using a Long-Short Term Memory (LSTM) network. They used both the ToN-IoT and InSDN datasets for training and testing. Their experimental results were 96.56% and 99.39% accuracy for the ToN-IoT and InSDN datasets, respectively. They also achieved 97.35% and 98.45% as F1 Score for ToN-IoT and InSDN datasets, respectively.

Y. Li et al.^28^ proposed a combining Transformer, federated learning, and Paillier cryptosystem method. They used the Transformer for detection, it was deployed in edge nodes. The proposed approach enables collaborative training of a detection model using data from all edge nodes by using a federated learning framework. The authors compare their results with a CNN and an LSTM model. The dataset was generated using IEEE 14-bus and IEEE 118-bus systems. The proposed model achieved an accuracy above 90% for both strong and weak attacks.

After investigating a wide range of research, we noted that most of the used datasets were built using conventional networks, and adapted to serve as SDN network datasets. The InSDN dataset represents an exception, as it was built using SDN traffic so it will be our concern. Additionally, our models will be based on the CNN-LSTM technique which appears to be a promising technique, and another model based on the Transformer architecture, the most significant and popular DL technique today.

## Background

Deep learning (DL) approaches have evolved from machine learning (ML) ones. In traditional ML, developers must manually choose the most important features or apply further analysis. Then, the models automatically figure out how to translate the features into outputs. On the other hand, DL constructs the features automatically in a multi-level process, starting with some chosen features or the whole possible set of features. Then, while going through the process, each level will contract some abstract features and feed them to the next level until reaching the output.

### Convolutional Neural Network Long Short-Term Memory (CNN-LSTM)

**Fig. 1:**
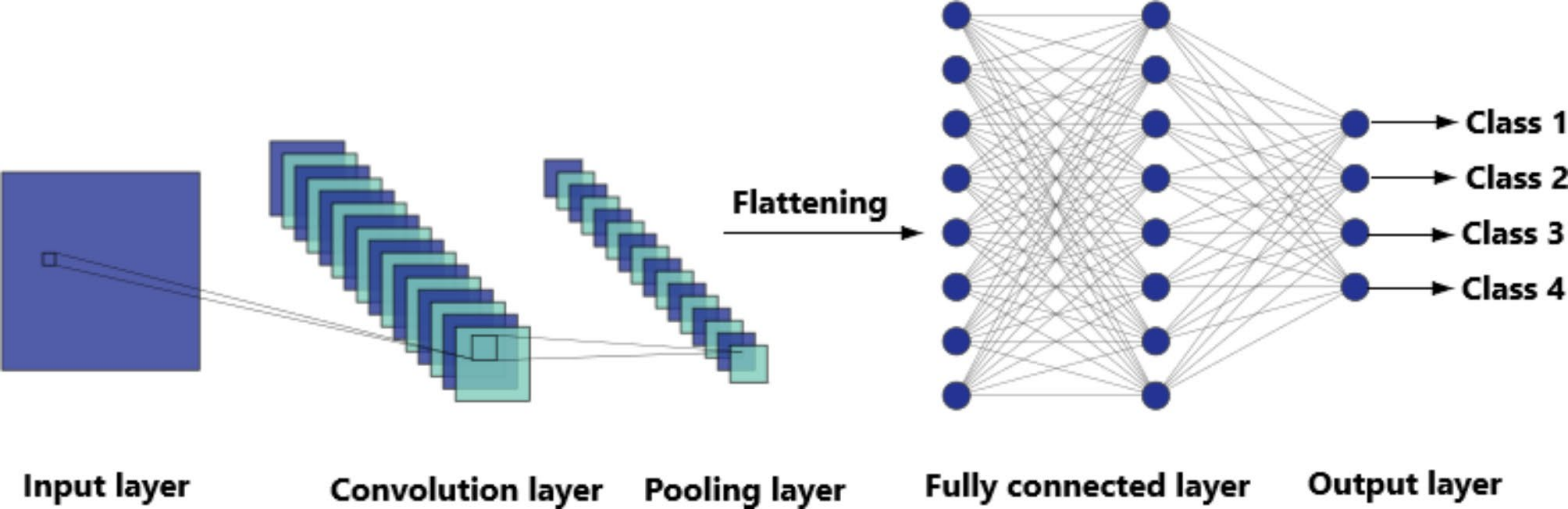
CNN architecture.

First, convolutional neural networks (CNN), which considered the most important ML technique in the realm of image processing and later on in almost any classification problem. One of the advantages of CNNs is their ability to learn features from the data without using any feature extraction methods. Another advantage is the ability to find more features out of the produced features. The network learns to identify features through the back-propagation process. A typical CNN architecture Fig. 1 consists of a series of layers:

- **Input Layer**: Represents the received inputs, it has a neuron for each number in the input data.
- **Convolution (CONV) Layer**: This layer applies a set of learnable filters to the input. Each filter activates certain features from the input, and by changing the number of filters and filter size in each layer we can construct different features.
- **Activation Layer**: Often we use Rectified Linear Unit (ReLU) as an activation function. Its mission is to introduce non-linearity into the network, allowing it to learn more complex features.
- **Pooling (POOL) Layer**: This layer decreases the number of parameters, which is responsible for decreasing the model computations, by reducing the spatial size of the CONV layers.
- **Fully Connected Layer**: It’s the final layer before the output layer. It is built using a fully connected network of neurons. It’s responsible for classifying the features learned by the CONV layers into the output classes.
- **Output Layer**: This layer is crucial for producing the final results after processing the input through the previous layers. It typically consists of neurons that represent the classes for a classification task.

Second, Long short-term memory (LSTM), which considered another type of deep learning (DL) networks. Unlike CNN, It has a feedback architecture. It is an evolution of recurrent neural networks (RNN), which solve the problem of vanishing gradient. It can also learn long-term dependencies between different data records during the training process.

**Fig. 2:**
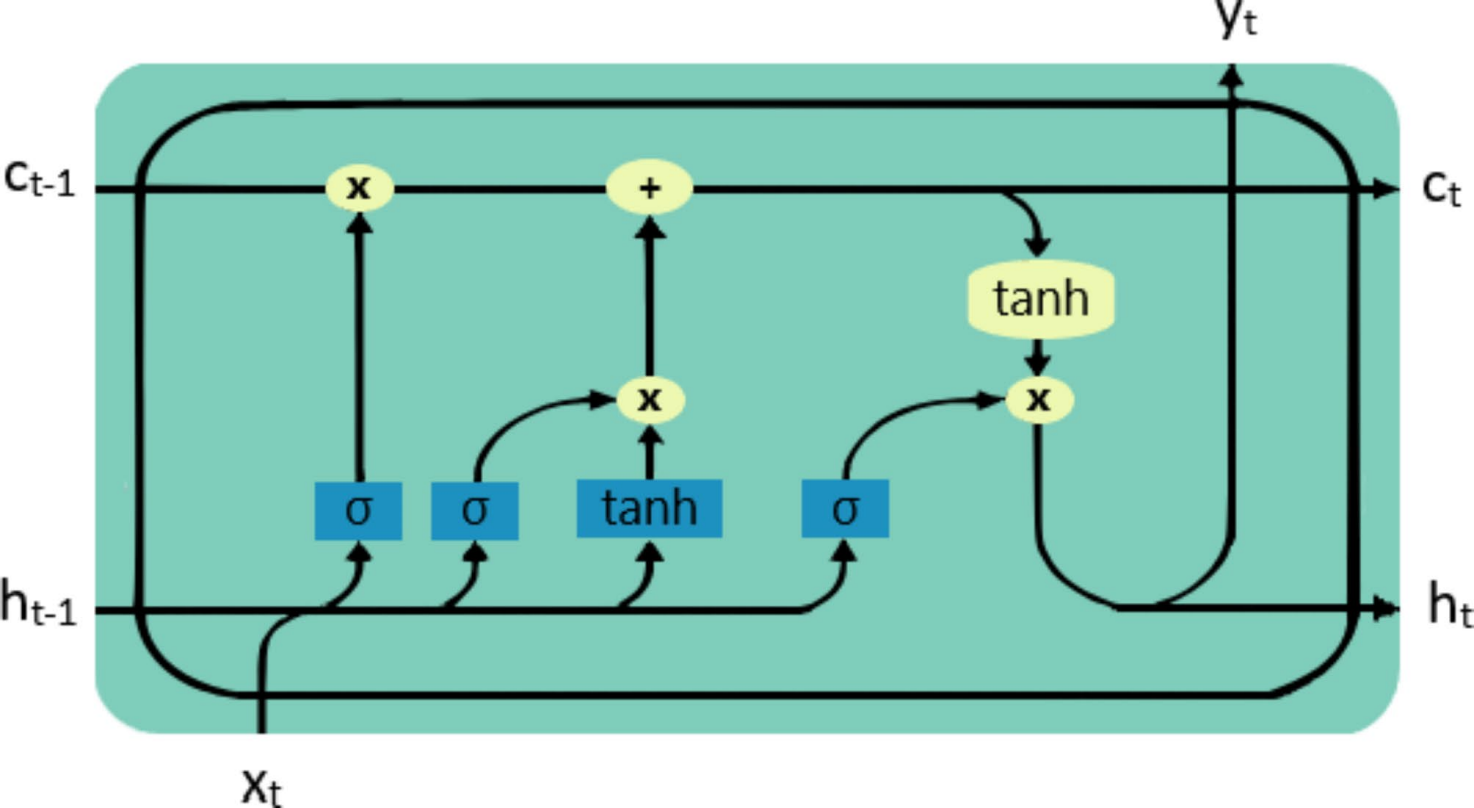
An LSTM cell.

The construction units of an LSTM network are called cells Fig. 2, the cell itself has three types of gates input, output, and forget gates. The purpose of the gates is to regulate the flow of information, so they typically choose to keep or discard this information. Forget gate Eqation 1 decides which information is discarded from the cell, where σ represents the sigmoid function, W_f is the weight matrix for the forget gate, b_f is the bias term, h_{t-1} is the previous hidden state, and x_t is the current input vector. Input gate Eqation 2 decides which values to be updated in the candidate cell state Eqation 3, where W_i and W_C are the weight matrices b_i, and b_C are the basis terms for the input gate and candidate cell state respectively. The cell state is updated by forgetting the old state and adding the new candidate values Eqation 4. Finally, the output gate Eqation 5 decides what the next hidden state Eqation 6 is, where W_o is the weight matrix, and b_o is the bias term for the output gate. The * operator represents element-wise multiplication, and tanh function output values between − 1 and 1 to help regulate the network’s internal state.

1 $$\:{f}_{t}=\sigma\:({W}_{f}\cdot\:[{h}_{t-1},{x}_{t}]+{b}_{f})$$

2 $$\:{i}_{t}=\sigma\:({W}_{i}\cdot\:[{h}_{t-1},{x}_{t}]+{b}_{i})$$

3 $$\:{\stackrel{\sim}{C}}_{t}=\text{t}\text{a}\text{n}\text{h}({W}_{C}\cdot\:[{h}_{t-1},{x}_{t}]+{b}_{C})$$

4 $$\:{C}_{t}={f}_{t}*{C}_{t-1}+{i}_{t}*{\stackrel{\sim}{C}}_{t}$$

5 $$\:{o}_{t}=\sigma\:({W}_{o}\cdot\:[{h}_{t-1},{x}_{t}]+{b}_{o})$$

6 $$\:{h}_{t}={o}_{t}*\text{t}\text{a}\text{n}\text{h}\left({C}_{t}\right)$$

### Transformer

Transformer-based models come with a great impact on the field of deep learning. At the heart of these models, we have self-attention mechanisms that provide a novel architecture and avoid traditional sequential processing in favor of parallelized one. The Transformer architecture was first introduced in Vaswani et al. paper “Attention Is All You Need”^29^. The new architecture introduces the idea of parallel processing in the scope of Machine Learning (ML) modeling. Unlike traditional algorithms such as neural networks (RNNs) and convolutional neural networks (CNNs), that use sequence modeling.

The self-attention mechanism, which is the core of the Transformer architecture enables Transformers to capture long-range dependencies and patterns. Transformers typically consist of an encoder and a decoder component, by permuting these components we can obtain more than one Transformer architecture such as encoder-only, encoder-decoder, and decoder-only Transformer. In this paper, we chose to work with encoder-only architecture, which is considered to be the most suitable one for classification. The first step involves encoding the input, this step aims to summarize the input information by representing each feature as a high-dimensional vector through an embedding layer. Transformer-based models often employ multi-head attention to reinforce the power of the self-attention mechanism. The process projects the input into multiple subspaces, which allow the model to attend to different parts of the input simultaneously. The transformer architecture typically includes feed-forward neural networks (FFNNs) in each layer. These FFNNs introduce non-linearity to the model and enable it to learn complex relationships between features. The outputs of the FFNNs are then passed through another layer normalization step. Finally, the transformer workflow involves generating output classes. This is typically done using a softmax layer.

## Proposed models

In this paper, we propose and compare two distinct models for network intrusion detection. The first is a hybrid architecture combining Convolutional Neural Networks (CNN) and Long Short-Term Memory (LSTM) networks, and the other utilizes a Transformer encoder-only architecture. We can list our contributions as the following:

- Build the CNN-LSTM model, which managed us to capture local patterns using CNN layers and long-term dependencies using LSTM layers.
- Build the Transformer model, which allowed us to efficiently process inputs in parallel and capture long-range dependencies.
- Train the models using multi-classification and binary classification.
- Efficiently reduce the training and testing set to only 4 features.

### CNN-LSTM proposed model

In the development of the CNN-LSTM model for network intrusion detection, several preprocessing steps were undertaken. Initially, the dataset was cleaned to remove any inconsistencies or outliers that could affect the model’s performance. Following cleaning, the data was shuffled to randomize the order of samples. Finally, the data underwent normalization using standard scaling. The standard scaling process involves subtracting the mean and dividing by the standard deviation for each feature, that effectively transforming the data to have a mean of zero and a standard deviation of one Eq. 7. After normalization, the input data was reshaped from its original format of 48 by 1 to a more suitable format of 6 by 8, which is compatible with the subsequent CNN layers.

7 $$\:{x}_{scaled}=\frac{x-mean\left(x\right)}{std\left(x\right)}$$

**Fig. 3:**
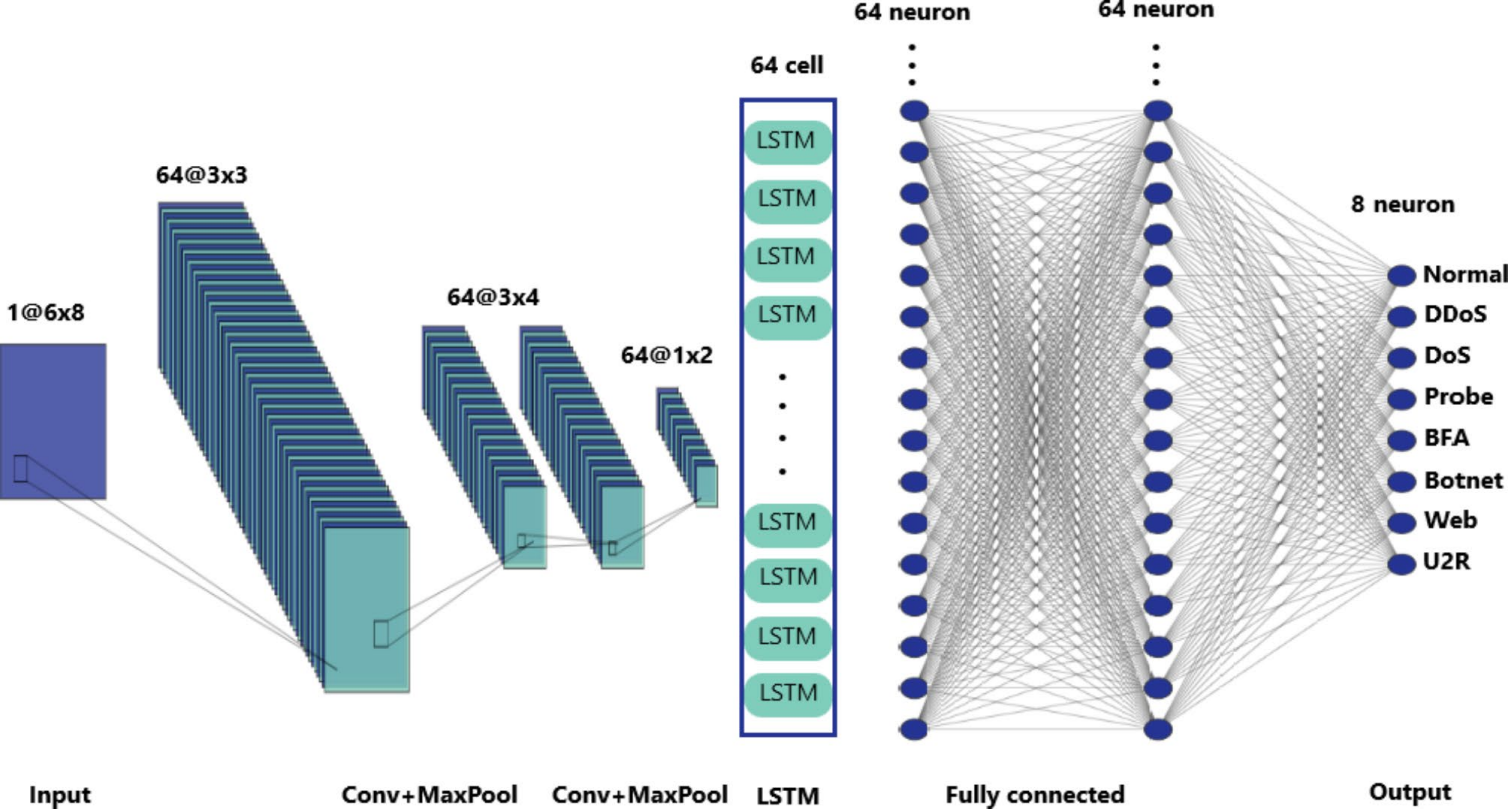
Proposed CNN-LSTM model architecture.

The CNN-LSTM model architecture Fig. 3 comprised two convolutional layers followed by two max-pooling layers. The convolutional layers will help us capture local patterns and features by utilizing learnable filters to convolve over the input data. Subsequently, max-pooling layers were employed to downsize the feature maps. Following the convolutional layers, a single LSTM layer was incorporated into the model architecture. The LSTM layer played a crucial role in capturing learning long-term dependencies. After the LSTM layer, the network architecture included two dense layers. The purpose of these layers is to calculate the model weights, which will be used later for the classification process. Finally, a softmax layer was added to produce probabilistic predictions across the different classes.

### Transformer model

In the development of the Transformer model, preprocessing steps similar to those of the CNN-LSTM model were undertaken. In the beginning, the dataset was cleaned to remove any inconsistencies or outliers. Then, the dataset was shuffled to randomize the order of samples. Lastly, it was normalized using standard scaling to standardize the features. Unlike the CNN-LSTM model, reshaping the input data was unnecessary for the Transformer model. Transformers’ self-attention mechanism can process input sequences without the need for reshaping.

**Fig. 4:**
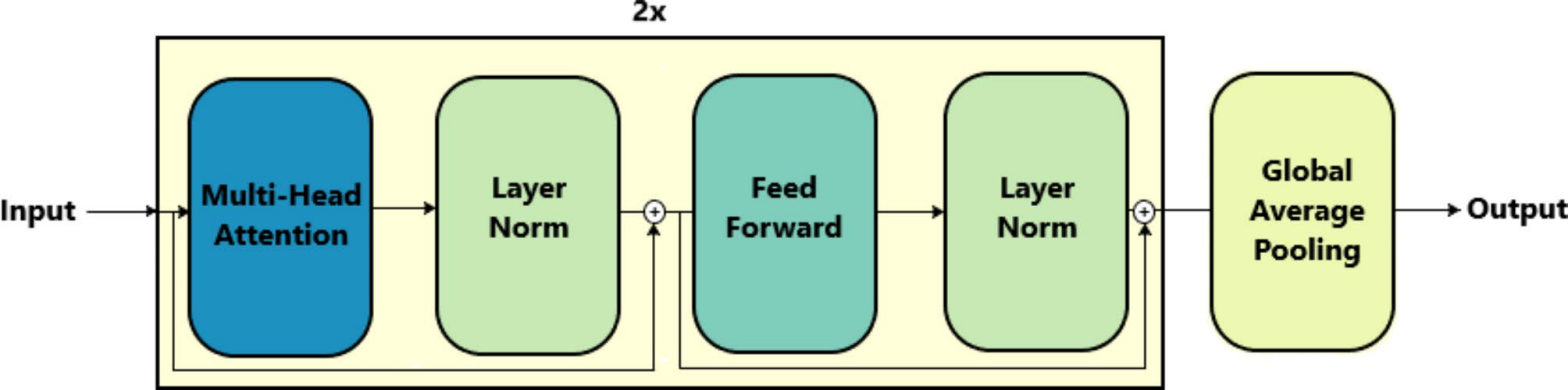
Proposed Transformer model architecture.

The Transformer model architecture Fig. 4 comprised a decoder-only design for our classification task. The architecture began with a multi-headed self-attention layer with two heads. These heads enable the model to capture dependencies between different parts of the input sequence. Following the self-attention layer, a normalization layer was applied to stabilize the learning process. Subsequently, the architecture included five one-dimensional convolutional layers (conv1d layers), each followed by normalization itself. These convolutional layers served to further extract features from the input sequence. That enhances the model’s ability to discern patterns relevant to network intrusion detection. After the convolutional layers, a global average pooling 1D layer was incorporated into the architecture. The model concluded with a softmax dense layer at the end, just like the CNN-LSTM model.

## Results and evaluation

In this section, we introduce a comprehensive evaluation of our models, the dataset we chose for training and testing, and the results we obtained.

### Dataset

Numerous datasets have been developed for training network-based machine learning models. The InSDN^30^ dataset is a crucial resource in Software Defined Networking (SDN). It offers precious insights into network traffic behavior. As the authors mentioned, this dataset is derived from real-world network traffic in an SDN environment. It contains a comprehensive repository of packet-level information. It includes various network activities such as host communications, flow characteristics, and potential security incidents. The dataset effectively captures the complexities of SDN infrastructures and facilitates in-depth exploration of SDN deployment.

The InSDN dataset includes a wide range of network attacks along with normal traffic records with a total size reach to 343,889 records, as detailed in Table 1. Each record originally consisted of 77 raw features after removing socket information like IP addresses, flow IDs, etc. These features were subsequently reduced to 48 by other researchers, and further to 9 features. In our study, we refined the feature set even further, we were able to reduce it to 6 features as detailed in Table 2, and finally to only 4 features in Table 3.

**Table 1:** InSDN classes list.

Class Name	Number of records
Normal DoS (Denial of Service)	68,424 53,616
DDoS (Distributed DoS) probe brute-force-attack Exploitation(r2l) web_attack	121,942 98,129 1405 17 192
Botnet Activity	164

**Table 2:** InSDN 6-features.

No.	Features Name	NO.	Features Name
1 2 3	Bwd Header Len Flow Duration Fwd Header Len	**4** **5** **6**	Flow Byts/s Pkt Len Std Pkt Size Avg

**Table 3:** InSDN 4-features.

No.	Features Name	NO.	Features Name
1 2	Bwd Header Len Flow Duration	**3** **4**	Flow Byts/s Pkt Len Std

### Evaluation metrics

To assess our DL models, we tried some of the most famous evaluation metrics such as accuracy, precision, recall, and F1 score. These metrics provided us with a comprehensive understanding of our models’ performance in classifying both malicious and benign network traffic. As known the calculation of these metrics includes the four special values of the confusion matrix, which are:

- **True Positives (TP)**: Represent the correctly identified malicious records.
- **True Negatives (TN)**: Represent the correctly identified benign records.
- **False Positives (FP)**: Represent the incorrectly identified malicious records.
- **False Negatives (FN)**: Represent the incorrectly identified benign records.

**Accuracy** measures the proportion of correctly predicted records out of the total instances.

8 $$\:Accuracy=\frac{TP+TN}{TP+TN+FP+FN}$$

**Precision** measures the proportion of correctly predicted positive records out of the total predicted positive record.

9 $$\:Precision=\frac{TP}{TP+FP}$$

**Recall** measures the proportion of correctly predicted positive records out of the total actual positive records.

10 $$\:Recall=\frac{TP}{TP+FN}$$

**The F1 score** provides a single metric that balances the model’s precision and recall.

11 $$\:F1\:Score=\frac{2\:\times\:\:Precision\:\times\:\:Recall}{Precision\:+\:Recall}$$

### Experimental results

The results presented in this section were obtained using a laptop with Windows 10 Pro, a Core i5-9300 H CPU, a GTX 1650 GPU, and 8 GB of RAM. This hardware configuration was employed to train and evaluate our models on the InSDN dataset. We used accuracy, precision, recall, and F1-score to assess the ability of each model to classify network traffic into the 8 classes mentioned in Table 1. We also, experimented with different numbers of features 48 features, 6 features, and 4 features. In Table 4, we present the summarized results of our experiments.

**Table 4:** Obtained results for both the CNN-LSTM and Transformer using 8 classes.

Num Of Features	Model	Accuracy	Precision	recall	f1 score
*48 features*	CNN-LSTM Transformer	99.01% 99.02%	99.03% 99.05%	99.01% 99.00%	99.02% 99.02%
*6 features*	CNN-LSTM Transformer	98.80% 98.90%	98.90% 98.98%	98.80% 98.85%	98.85% 98.91%
*4 features*	CNN-LSTM Transformer	98.20% 98.50%	98.20% 98.52%	98.20% 98.47%	98.20% 98.49%

The Transformer model with 48 features achieved the highest accuracy at 99.02%. The CNN-LSTM one with the same number of features followed closely, achieving an accuracy of 99.01%, with the same F1 score percentages for both we cannot prefer one model over the other. These results indicate that the models perform well with a comprehensive set of features. When the number of features was reduced, the Transformer model with 6 features maintained a high accuracy of 98.90% against 98.80% for the CNN-LSTM. With another decaying in the features set to be only 4 features, the Transformer model proves its superiority with 98.50% accuracy, slightly lower but still significantly high compared with using the whole set of features. Detailed results are shown in Table 5.

**Table 5:** Detailed results for CNN-LSTM and Transformer using 8 classes.

48 features									
*Model*	*METRIC*	*NORMAL*	*DDOS*	*DOS*	*PROBE*	*BOTNET*	*BFA*	*U2R*	*WEB-ATTACK*
CNN-LSTM	Accuracy Precision Recall F1 Score	99.22% 99.43% 96.61% 98.00%	99.99% 100% 99.99% 99.99%	99.82% 99.60% 99.22% 99.41%	99.19% 97.45% 99.81% 98.62%	99.99% 93.33% 77.78% 84.85%	99.86% 83.98% 78.75% 81.29%	100% 100% 40.00% 57.14%	99.96% 100% 35.90% 52.83%
Transformer	Accuracy Precision Recall F1 Score	99.16% 99.19% 96.60% 97.88%	99.99% 99.97% 100% 99.99%	99.81% 99.90% 98.91% 99.40%	99.23% 97.48% 99.89% 98.67%	99.99% 78.72% 100% 88.10%	99.89% 88.89% 79.01% 83.66%	100% 100% 100% 100%	99.98% 100% 55.88% 71.70%
6 features									
*Model*	***METRIC***	***NORMAL***	***DDOS***	***DOS***	***PROBE***	***BOTNET***	***BFA***	***U2R***	***WEB-ATTACK***
CNN-LSTM	Accuracy Precision Recall F1 Score	99.17% 99.41% 96.37% 97.86%	99.99% 99.98% 99.99% 99.98%	99.69% 98.98% 98.99% 98.99%	99.19% 97.42% 99.83% 98.61%	99.98% 100% 58.33% 73.68%	99.66% 57.71% 53.48% 55.51%	99.99% 100% 20.00% 33.33%	99.95% 100% 5.13% 9.76%
Transformer	Accuracy Precision Recall F1 Score	99.18% 99.53% 96.37% 97.93%	99.98% 99.94% 100% 99.97%	99.75% 99.14% 99.29% 99.21%	99.19% 97.45% 99.78% 98.60%	99.98% 100% 59.46% 74.58%	99.75% 66.35% 56.79% 61.20%	100% 100% 100% 100%	99.97% 93.75% 44.12% 60.00%
4 features									
*Model*	***METRIC***	***NORMAL***	***DDOS***	***DOS***	***PROBE***	***BOTNET***	***BFA***	***U2R***	***WEB-ATTACK***
CNN-LSTM	Accuracy Precision Recall F1 Score	98.71% 98.97% 94.45% 96.66%	99.93% 99.85% 99.96% 99.90%	99.17% 95.94% 98.78% 97.34%	99.08% 97.25% 99.62% 98.42%	99.98% 100% 58.33% 73.68%	99.68% 70.99% 34.07% 46.04%	99.99% 0% 0% 0%	99.94% 0% 0% 0%
Transformer	Accuracy Precision Recall F1 Score	98.80% 98.53% 95.43% 96.96%	99.97% 99.93% 99.99% 99.96%	99.44% 98.03% 98.36% 98.20%	99.10% 97.26% 99.69% 98.46%	99.98% 100% 59.46% 74.58%	99.76% 71.74% 54.32% 61.83%	99.99% 0% 0% 0%	99.95% 0% 0% 0%

**Fig. 5:**
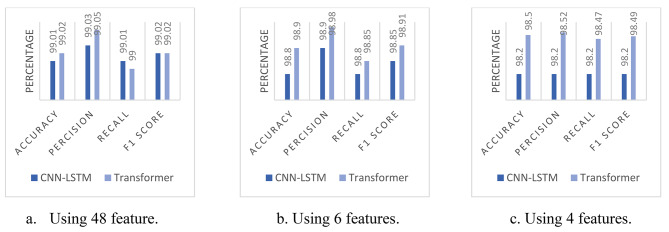
Comparing results for both models using 8 classes.

When evaluating the performance of the CNN-LSTM and Transformer models using 48 features for each attack type, we observed that the results for normal traffic, DDoS, DoS, and probe attacks were satisfying across all four metrics. The models achieved good accuracy, precision, recall, and F1 scores for these attack types, as shown in Fig. 5.

**Fig. 6:**
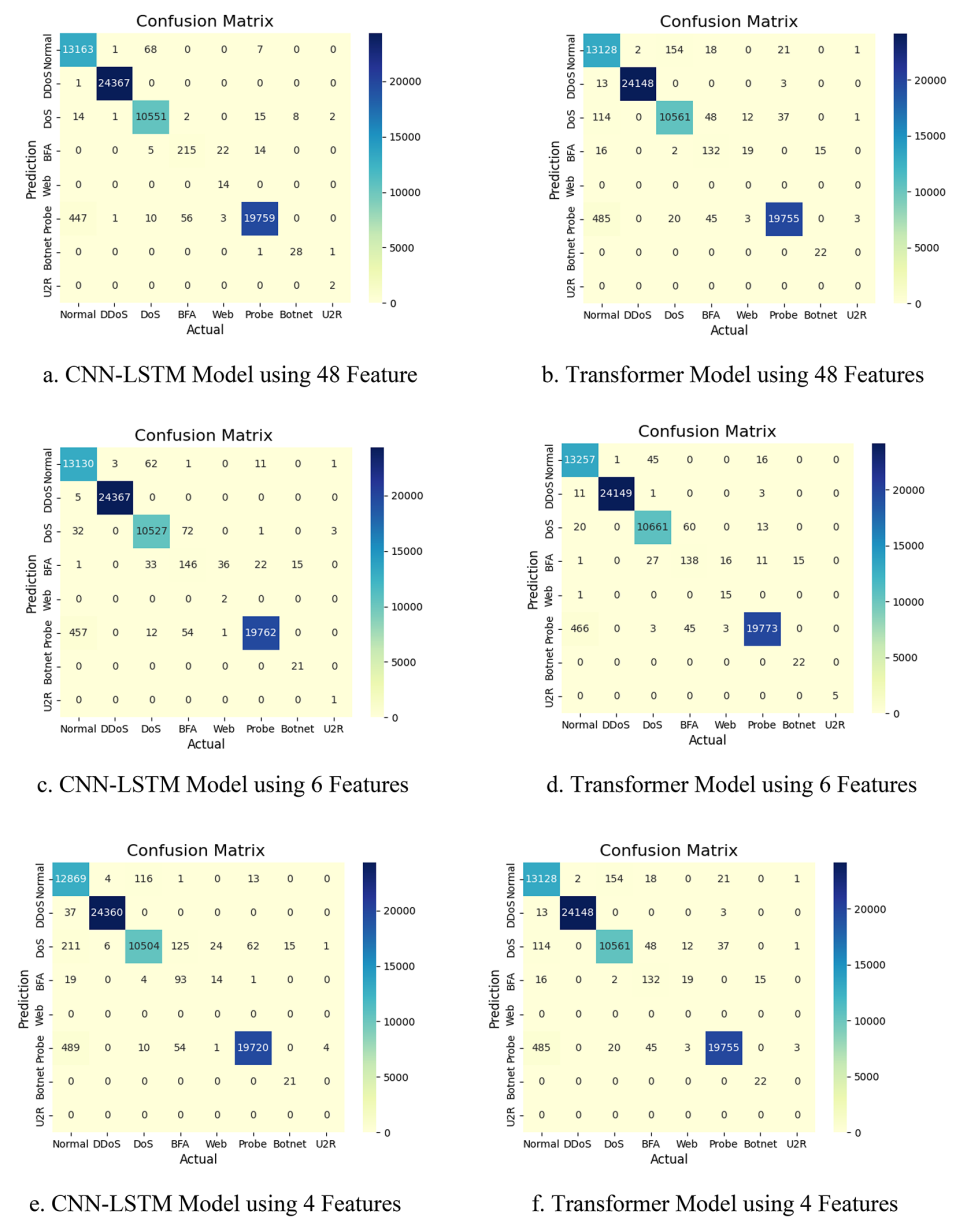
Confusion matrices for both CNN-LSTM and Transformer.

However, the performance for brute force attack (BFA), web-attack, botnet, and user-to-root (U2R) attacks was significantly lower according to the results in Table 5. This poor performance can be attributed to the limited number of records available for these attack types in the dataset. We have only 17 records for U2R, 164 for botnet, 192 for web-attack, and 1405 for BFA. The scarcity of training data for these attacks likely hindered the models’ ability to learn patterns. That causes many misclassifications in these categories, which appears in the confusion matrix Fig. 6.

**Fig. 7:**
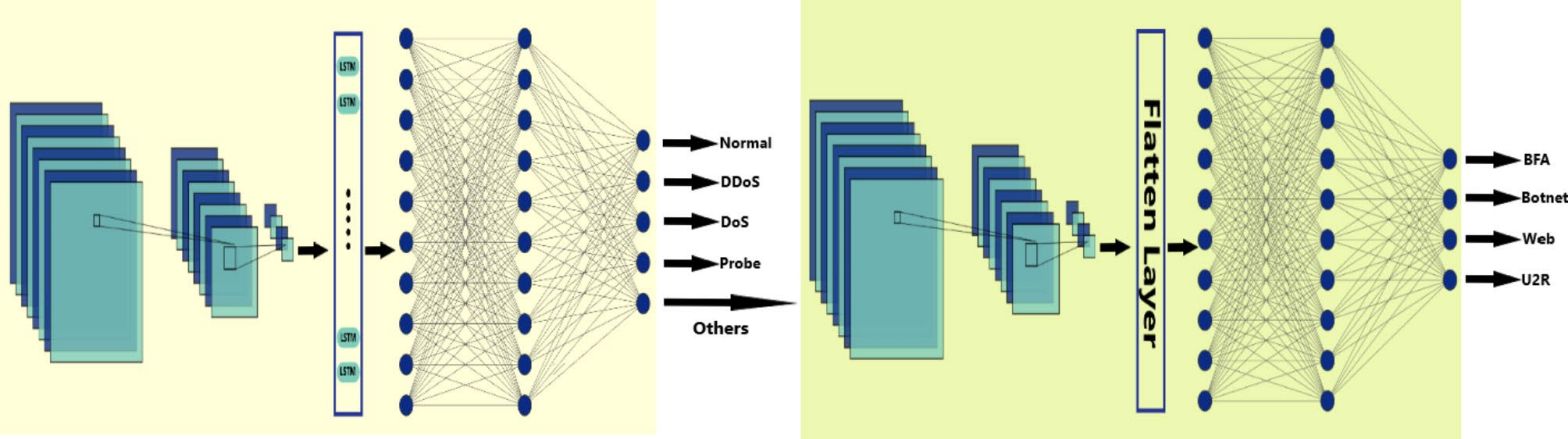
Two stages model for classifying others.

To solve the problem of low accuracy for BFA, U2R, web attacks, and botnet attacks, we decided to group them into one class called **Others**, hence we have 5 classes. The results of this experiment showed an improvement at all levels except for the 4 features set, as shown in Tables 6 and 7. Furthermore, we upsampled U2R, web attacks, and botnet attacks to be 1405 records, the same as BFA, and then we built another classifier specially for these 4 attacks Fig. 7. The special classifier was a simple CNN model, the results for this classifier were excellent and beyond expected, as shown in Table 8.

**Table 6:** Obtained results for both the CNN-LSTM and Transformer using 5 classes.

Num Of Features	Model	Accuracy	Precision	recall	f1 score
*48 features*	CNN-LSTM Transformer	99.02% 99.12%	99.04% 99.13%	99.02% 99.12%	99.03% 99.12%
*6 features*	CNN-LSTM Transformer	98.89% 99.03%	98.91% 99.04%	98.88% 99.01%	98.89% 99.02%
*4 features*	CNN-LSTM Transformer	98.41% 98.36%	98.42% 98.39%	98.40% 98.33%	98.41% 98.36%

**Table 7:** Detailed results for CNN-LSTM and Transformer using 5 classes.

48 features						
*Model*	*METRIC*	*NORMAL*	*DDOS*	*DOS*	*PROBE*	*OTHERS*
CNN-LSTM	Accuracy Precision Recall F1 Score	99.19% 99.76% 96.13% 97.91%	99.99% 99.99% 99.99% 99.99%	99.81% 99.19% 99.55% 99.37%	99.20% 97.42% 99.87% 98.63%	99.86% 91.37% 81.025 85.89%
Transformer	Accuracy Precision Recall F1 Score	99.42% 99.59% 96.61% 98.08%	100% 99.99% 100% 99.99%	99.89% 99.87% 99.41% 99.64%	99.22% 97.46% 99.90% 98.66%	99.90% 95.00% 83.39% 88.81%
6 FEATURES						
*Model*	***METRIC***	***NORMAL***	***DDOS***	***DOS***	***PROBE***	***OTHERS***
CNN-LSTM	Accuracy Precision Recall F1 Score	99.19% 99.48% 96.40% 97.92%	99.99% 99.98% 99.99% 99.99%	99.70% 98.92% 99.15% 99.04%	99.19% 97.47% 99.77% 98.60%	99.72% 79.21% 62.61% 69.94%
Transformer	Accuracy Precision Recall F1 Score	99.18% 99.53% 96.37% 97.92%	99.98% 99.95% 100% 99.97%	99.84% 99.67% 99.29% 99.48%	99.20% 97.46% 99.82% 98.63%	99.86% 86.32% 83.07% 84.66%
4 FEATURES						
*Model*	***METRIC***	***NORMAL***	***DDOS***	***DOS***	***PROBE***	***OTHERS***
CNN-LSTM	Accuracy Precision Recall F1 Score	98.84% 98.64% 95.47% 97.03%	99.98% 99.99% 99.95% 99.97%	99.31% 97.21% 98.37% 97.79%	99.07% 97.11% 99.74% 98.41%	99.62% 83.09% 32.01% 46.22%
Transformer	Accuracy Precision Recall F1 Score	98.77% 98.64% 95.16% 96.87%	99.93% 99.81% 100% 99.90%	99.26% 97.29% 98.01% 97.65%	99.10% 97.25% 99.71% 98.47%	99.66% 73.89% 41.69% 53.31%

**Table 8:** Detailed results for others classes using CNN model.

48 features					
*Model*	***METRIC***	***BFA***	***WEB-ATTACK***	***BOTNET***	***U2R***
CNN	Accuracy Precision Recall F1 Score	100% 100% 100% 100%	100% 100% 100% 100%	100% 100% 100% 100%	100% 100% 100% 100%
6 features					
*Model*	***METRIC***	***BFA***	***WEB-ATTACK***	***BOTNET***	***U2R***
CNN	Accuracy Precision Recall F1 Score	99.90% 99.66% 100% 99.83%	99.90% 100% 99.59% 99.79%	99.62% 99.16% 99.16% 99.16%	99.81% 99.64% 99.64% 99.64%
4 features					
*Model*	***METRIC***	***BFA***	***WEB-ATTACK***	***BOTNET***	***U2R***
CNN	Accuracy Precision Recall F1 Score	99.71% 98.97% 100% 99.48%	99.81% 100% 99.15% 99.57%	98.76% 95.56% 99.61% 97.54%	98.67% 99.61% 95.13% 97.32%

The improvements in the results of the 5-classes classification have inspired us to perform a binary classification by merging all attack types into a single class, the results are shown in Table 9. This approach improved results for both models. Using 48 features, the accuracy for the CNN-LSTM model increased to 99.08%, and for the Transformer model, it increased to 99.16%. Remarkably, with 6 features, the CNN-LSTM model achieved an accuracy of 99.19%, surpassing its performance with 48 features. This result was unexpected and suggests that the model may be more efficient with a streamlined feature set. The Transformer model showed no improvement with 6 features. With 4 features, the CNN-LSTM model reached an accuracy of 98.86%, while the Transformer model improved significantly to 98.93%.

**Table 9:** Experimental results for both the CNN-LSTM and Transformer using binary classification.

Num Of Features	Model	Accuracy	Precision	recall	f1 score
*48 features*	CNN-LSTM Transformer	99.08% 99.16%	99.08% 99.16%	99.08% 99.16%	99.08% 99.16%
*6 features*	CNN-LSTM Transformer	99.19% 99.16%	99.19% 99.16%	99.19% 99.16%	99.19% 99.16%
*4 features*	CNN-LSTM Transformer	98.86% 98.93%	98.86% 98.93%	98.86% 98.93%	98.86% 98.93%

**Fig. 8:**
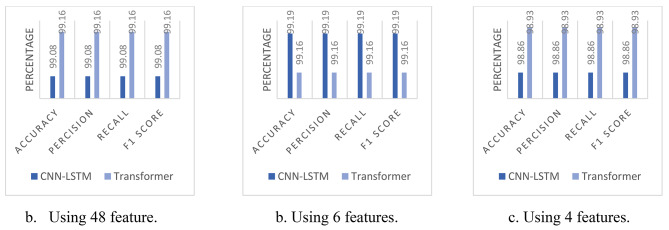
Comparing results for both models using binary classification.

Binary classification has improved the accuracy and F1 score for both models Fig. 8. The higher metrics come from reducing the complexity of distinguishing between multiple attack types. However, multi-class classification is more valuable for most security applications. Where specifying the type of attack allows for more targeted mitigation strategies.

### Results discussion

**Fig. 9:**
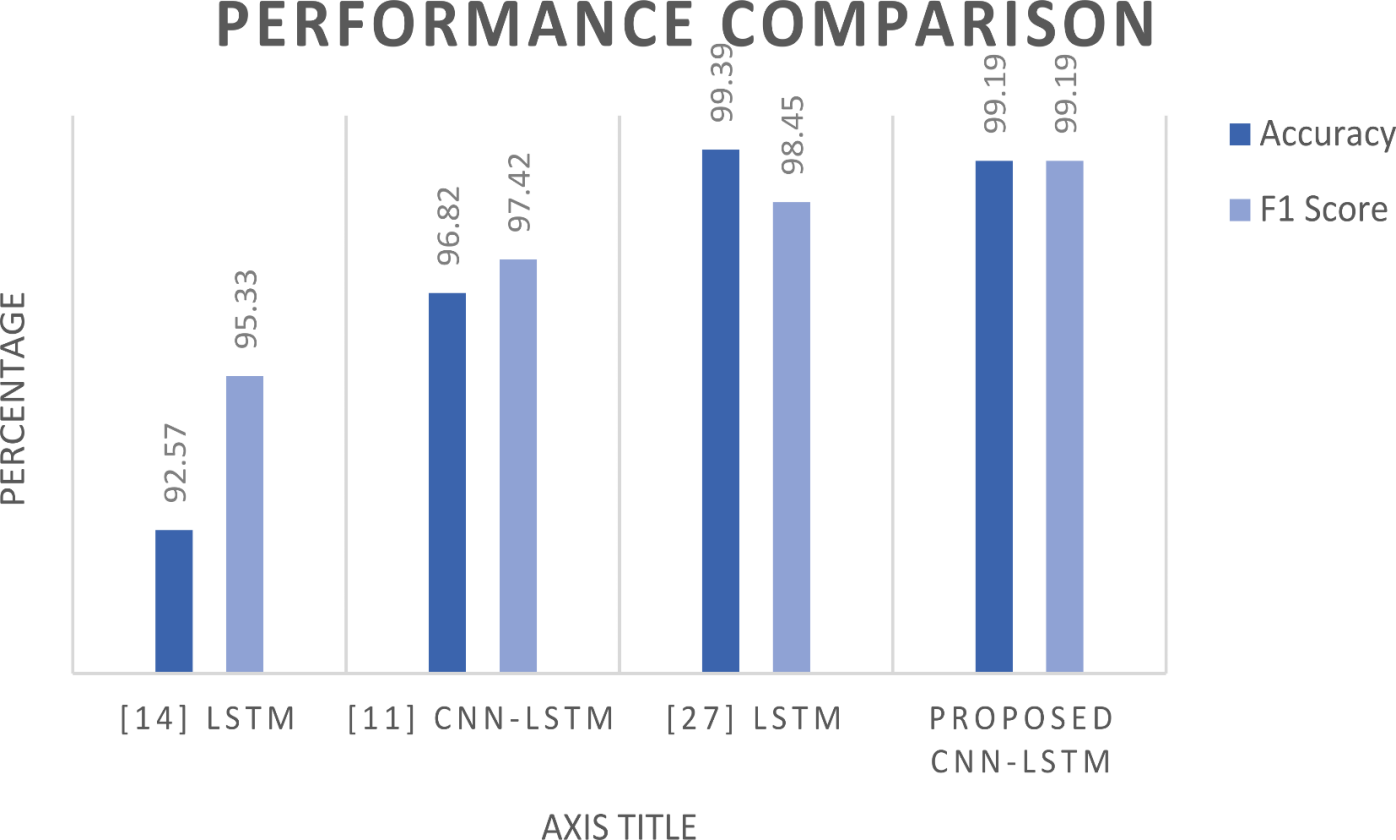
Comparing our results with related works that use the InSDN dataset.

In this section, we compare the performance of our proposed CNN-LSTM and Transformer models with those from related works. We examine the accuracy and F1 score metrics to get insights about these different models. It wasn’t easy to set such a comparison because most of the related works use different datasets and different sets of features. However, we tried to compare our CNN-LSTM with the works that use the InSDN dataset Fig. 9. The comparison that appears in Table 10 demonstrates that our CNN-LSTM model with 6 features has only 0.2% less than^27^ which trained using 48 features and 0.73% higher in F1 score, and beat all the others. For, the Transformer model, we are the first group to apply this technique to the InSDN dataset.

**Table 10:** Comparing our proposed models’ results with other state-of-the-art approaches.

No	Reference	Year	Model	Dataset	features	accuracy	f1 score
*1*	[12]	2019	GRU-RNN	NSL-KDD CICIDS2017	6 9	89.00% 99.00%	89.00% 99.00%
*2*	[13]	2020	LSTM	CICIDS2018	80	96.79%	-
*3*	[14]	2021	RNN LSTM GRU	InSDN	6	91.11% 92.57% 91.31%	94.51% 95.33% 94.62%
*4*	[15]	2021	Trans-CNN	CICDoS2019	-	99.82%	99.92%
*5*	[11]	2021	CNN-LSTM	InSDN	48	96.82%	97.42%
*6*	[25]	2021	RNN-LSTM	Their Own	7	89.63%	-
*7*	[16]	2022	Transformer	CICID2017 CICDDoS2019	79 86	99.53% 98.58%	99.17% 98.48%
*8*	[28]	2022	Transformer	Their Own	-	> 90.0%	76.03 − 100%
*9*	[18]	2023	BiLSTM	CICIDS2018 Edge_IIoT	77 62	100% 99.64%	- -
*10*	[19]	2023	MiniRocket	CICDDoS2019 CICIDS2018	- -	- -	99.95% 99.90%
*11*	[20]	2023	GAN-RNN	CICIDS2017	-	99.4%	99.4%
*12*	[21]	2022	LSTM	KDD_cup99	41	95.4%	95.1%
*14*	[22]	2023	LSTM-AE	CICIDS2017 CICIDS2018	81 78	99.99% 99.10%	99.99% 99.02%
*15*	[23]	2023	AE-LSTM	NSL-KDD	41	98.88%	98.70%
*16*	[24]	2023	CNN-LSTM	NSL-KDD	41	99.31%	99.18%
*17*	[26]	2023	Transformer	Their Own	256	96.50%	96.20%
*18*	[27]	2023	LSTM	InSDN ToN-IoT	48 43	99.39% 96.56%	98.45% 97.35%
*19*	[17]	2024	Transformer	CICIDS 2018	78	98.11%	65.18%
*20*	Proposed	This paper	CNN-LSTM	InSDN	48 6 4	99.08% 99.19% 98.86%	99.08% 99.19% 98.86%
*21*	Proposed	This paper	Transformer	InSDN	48 6 4	99.16% 99.16% 98.93%	99.16% 99.16% 98.93%

- Not mentioned.

## Conclusion

In this study, we developed and evaluated two distinct models for network intrusion detection a hybrid CNN-LSTM model and a Transformer encoder-only model. Both models were trained and tested using the InSDN dataset. We leveraged different numbers of features to assess their performance. Our evaluation metrics included accuracy, precision, recall, and F1 score, comprehensively assessing each model’s classification capabilities.

The Transformer model with 48 features achieved the highest accuracy. The CNN-LSTM model also performed well with 48 features, achieving a slightly lower accuracy. However, the CNN-LSTM model with 6 features overcomes the Transformer model. In contrast, both models maintained high accuracy even with fewer features. Both models are considered to be robust and capable of maintaining high performance with a reduced feature set. Another important metric that could be crucial is the training/testing time. The Transformer model has a significantly longer time, which needs to be considered when choosing between the models. This suggests that for our IDS application, where feature reduction and testing time are necessary. The CNN-LSTM model might be a more suitable choice.

When we evaluated the performance of these models for individual attack types using 48 features, we found that both models performed well for normal traffic, DDoS, DoS, and probe attacks. However, the performance was notably poorer for web attacks, botnet, BFA, and U2R attacks. We attribute that to the limited number of records available for these attack types. This pushed us to group all these poorly represented attacks in one group called Others and rerun the models. The models gave us better results, especially the Transformer. In addition, we create a CNN model specially for those 4 attacks to correctly reclassify a record if it was classified as Others by one of the original models.

Furthermore, we explored binary classification by merging all attack types into a single class. This approach yielded improved results for both models. But, it may not provide the granularity needed to distinguish between different types of attacks.

## Future work

In the near future, we will try to investigate several avenues. We may explore advanced feature selection techniques on the raw InSDN WireShark files. We will include evaluations on larger datasets and consider metrics such as training and testing time, computational cost, and learning curves. We also can include some real-world network traffic to provide a more comprehensive assessment. Another promising direction in the deep learning field is the ensemble learning techniques, which could help us to enhance the strength of both CNN-LSTM and Transformer models. We will try our best to develop reliable security solutions for software-defined networks.

## Data Availability

The datasets that support the findings of this research are available in “InSDN dataset files” at https://drive.google.com/drive/folders/16bRX1uo6zyKlkMgKqZDyc4DeYuBzfOxx?usp=sharing, reference number [31]. These data were derived from the following resources available in the public domain: http://iotseclab.ucd.ie/datasets/SDN/ or https://aseados.ucd.ie/datasets/SDN/.
